# Maternal Perception and Childhood Overweight: Examining Parenting Styles and Eating Behaviors Among Preschoolers: A Cross-Sectional Study from Samsun, Türkiye

**DOI:** 10.3390/nu17010195

**Published:** 2025-01-06

**Authors:** Refia Gözdenur Savcı, Sıddıka Songül Yalçın

**Affiliations:** 1 Alaçam State Hospital, 55800 Samsun, Türkiye; 2 Department of Social Pediatrics, Institute of Health Sciences and Institute of Child Health, Hacettepe University, 06230 Ankara, Türkiye

**Keywords:** maternal perception, childhood obesity, parenting styles, children’s eating behaviors, nurturing care

## Abstract

Background: Accurate maternal perceptions of children’s weight status are crucial for early childhood obesity prevention, with evidence suggesting that maternal misperception may delay timely interventions. This study investigated the accuracy of maternal perceptions of child weight and examined associations with parenting styles and children’s eating behaviors and demographic factors among preschool-aged children in Samsun, Türkiye. Methods: This cross-sectional study included 318 mother–child pairs recruited from preschools in socio-economically diverse areas of Samsun. Maternal perceptions of child weight status were assessed using a visual scale, while children’s anthropometric measurements (height, weight) were recorded to calculate BMI-for-age Z-scores. Parenting styles and children’s eating behaviors were evaluated using the Parental Attitude Scale (PAS) and the Children’s Eating Behavior Questionnaire (CEBQ), respectively. Data analysis involved chi-square tests and multiple logistic regression to explore associations between maternal perception accuracy, parenting styles, and children’s eating behaviors. Results: Among 318 mother–child pairs, 59.7% underestimated their child’s weight status, with this figure being 84.9% among overweight children. Authoritative parenting was the most common style (78%); no significant association was found between parenting style and maternal perception accuracy. Accurate estimators showed significantly higher scores for food responsiveness (median: 9 (7–12), *p* = 0.028) and the enjoyment of food (mean: 16.3 ± 4.8, *p* = 0.003), whereas underestimators scored higher for satiety responsiveness (mean: 23.2 ± 5.7, *p* = 0.042) and slowness in eating (mean: 11.2 ± 4.2, *p* = 0.004). Conclusions: Maternal underestimations of child weight are prevalent, particularly for overweight children, are associated with children’s specific eating behaviors. Targeted educational interventions focusing on improving maternal awareness and promoting responsive feeding practices are essential to combat childhood obesity.

## 1. Introduction

Childhood obesity is a critical global health issue, described by the World Bank as a “ticking time bomb”. Rising prevalence rates, driven by poor dietary habits and insufficient physical activity, have made it a pressing concern worldwide [1]. According to joint estimates from UNICEF and the World Health Organization, 6% of children under 5 years old and 18% of children aged 5–19 years old are overweight, accounting for over 380 million children globally [2]. In Türkiye, the situation is similarly concerning, with 8% of under-five children classified as overweight or obese [3]. Although specific data for Samsun are unavailable, the region’s socio-economic diversity provides a unique context for exploring childhood obesity, influenced by both Eastern and Western cultural norms. In Türkiye, the Ministry of Health’s programs are present to promote balanced nutrition and physical activity in early childhood [4]. However, these efforts rely heavily on parental awareness, particularly the maternal recognition of children’s weight status, to initiate preventive measures at home.

Maternal perception—specifically, the ability to recognize a child’s overweight status [5,6]—is pivotal in early obesity prevention. However, maternal misperception—failure to accurately assess a child’s weight [7,8]—frequently delays action, perpetuating unhealthy behaviors. Childhood obesity increases the risk of chronic diseases such as cardiovascular disease, diabetes, and cancer later in life [1,4].

While substantial research has investigated parental feeding practices, such as restrictive or pressuring behaviors [9,10,11,12], the relation between maternal perceptions and children’s eating behaviors remains understudied. Emotional eating, for instance, may be shaped by familial stressors, while food responsiveness reflects parental modeling [13]. Additionally, satiety responsiveness has been linked to consistent feeding practices that encourage self-regulation [14]. Mothers’ understanding of their children’s eating styles and weight status is crucial, as it directly affects their ability to implement appropriate nutritional decisions. Misjudging a child’s eating behaviors or body size can lead to overfeeding, underfeeding, or inappropriate dietary restrictions, potentially contributing to unhealthy weight trajectories or nutritional imbalances [9,10,12,15]. In addition, the Children’s Eating Behavior Questionnaire (CEBQ) provides valuable insights into children’s eating behaviors, including food responsiveness, emotional eating, and satiety responsiveness, all of which influence body weight outcomes [16].

Parenting styles—defined as the emotional climate in which child-rearing behaviors are expressed—may play a crucial role in shaping children’s eating behaviors and weight outcomes. Authoritative parenting, which balances warmth and control, is consistently associated with healthier body weight and eating behaviors, fostering self-regulation and autonomy in dietary choices. This parenting style promotes open communication about food preferences, encouraging healthier eating behaviors and body image perceptions in children [17,18,19]. Authoritarian parenting, defined by high control and low warmth, often leads to power struggles over food, prompting children to eat in response to external pressures rather than their internal cues. This approach has been linked to a higher risk of childhood obesity and problematic eating behaviors [18,19,20]. Permissive parenting, characterized by high warmth but low control, poses unique challenges due to its lack of structure in promoting healthy eating habits. Children raised by permissive parents often have greater freedom to consume unhealthy foods and may struggle with self-regulation [21,22]. Overprotective parenting, characterized by excessive involvement in children’s choices—including dietary decisions—can undermine autonomy and self-regulation, often resulting in unhealthy eating habits and distorted body image perceptions [20,21,23]. The Nurturing Care Framework emphasizes the importance of responsive feeding, encouraging parents to recognize and appropriately respond to their children’s hunger and satiety cues [24,25]. This approach not only fosters a deeper understanding of children’s eating behaviors but also supports healthy growth and enables timely nutritional interventions. While authoritative parenting naturally aligns with responsive feeding by promoting autonomy and self-regulation, other parenting styles—such as the authoritarian, permissive, and overprotective styles—may require tailored strategies to effectively integrate these principles [19,21,22,25].

This study is guided by Bronfenbrenner’s Ecological Systems Theory, the Bidirectional Parent–Child Interaction Model, and Social Comparison Theory to explore the dynamics between maternal perception and childhood obesity. Bronfenbrenner’s Ecological Systems Theory provides a broader framework to understand how environmental factors across multiple levels influence maternal perception and child health outcomes [26,27,28]. In Samsun, a region blending Eastern and Western cultural influences, this framework highlights how socio-economic diversity impacts the maternal recognition of weight status, with higher socio-economic status hypothesized to improve accuracy. The Bidirectional Parent–Child Interaction Model underscores the mutual influence between parents and children, where maternal feeding practices shape children’s eating behaviors, and these behaviors, in turn, affect maternal strategies. For instance, a child’s food responsiveness might prompt specific parental approaches, reinforcing patterns that influence the maternal perception of body size [11,29,30]. Finally, Social Comparison Theory offers insight into how maternal perceptions are shaped by comparisons with peers and societal norms. In Samsun, cultural ideals associating larger body sizes with health and prosperity may contribute to the underestimation of children’s weight status, delaying necessary interventions. By integrating these frameworks, this study explores how socio-cultural, familial, and individual factors interact to influence maternal perception, providing valuable insights for addressing childhood obesity in diverse contexts [31,32,33].

We hypothesize that mothers with higher socio-economic status, better education, less information taken from relatives, authoritative parenting styles, and no significant health conditions are more likely to accurately perceive their children’s body size. Conversely, mothers with authoritarian parenting styles are more prone to misperception. Additionally, children’s eating behaviors—as reported by mothers—can further influence mothers’ recognition of their children’s body size. This theoretical framework aligns with research showing that parenting styles and child feeding practices interact to shape children’s health outcomes [34,35]. We aim to address the gap by examining the alignment between child eating behaviors and mothers’ accurate estimation of their children’s body size among kindergarten-aged children in Samsun, Turkey, while also considering parenting styles, socio-economic factors, and maternal health status. The findings of this study will contribute to the achievement of Sustainable Development Goals (SDGs), particularly Goal 2 (Zero Hunger) and Goal 3 (Good Health and Well-being), by emphasizing early maternal awareness and culturally relevant interventions that promote healthy child growth and responsive feeding practices, thereby informing sustainable strategies for addressing childhood obesity [36].

## 2. Materials and Methods

### 2.1. Study Design

This cross-sectional survey was conducted between November and December 2023 in preschools in Samsun, Türkiye, and data analysis was performed in January–April 2024.

### 2.2. The Determination of the Study Sample

According to Turkish Statistical Institute data, 92,792 children aged 5–9 reside in Samsun, with approximately 20,000 estimated to be 5 years old [37]. To detect a condition prevalence of 50% with a margin of error of d = 0.05, the required sample size was calculated as 377. To account for potential non-participation and data entry errors, an additional 20% was added, leading to a target sample size of 452.

### 2.3. Participant Selection and Sampling

This study’s sample was stratified across five regions within Samsun, classified by the Socio-Economic Development Ranking Index (SEGE). Sample sizes for each region were calculated and weighted proportionally to the number of children in each region to ensure representativeness [38]. Stratified sampling was employed based on the SEGE, ensuring that each region’s socio-economic diversity was proportionally represented. Within each SEGE-defined region, public preschools were randomly selected for participant recruitment, further enhancing the representativeness of the sample.

Ethical approval was granted by the Samsun University Clinical Research Ethics Committee, and permissions, along with a list of preschools and enrolment numbers, were obtained from the Samsun Provincial Directorate of National Education. Children aged 60–72 months old attending these institutions were then grouped according to SEGE-defined regions. For regions comprising multiple districts, a random sampling approach using a random numbers table was applied to select districts for this study. Within each selected district, data collection began at schools with the highest child enrollment. If the target sample size was unmet, additional schools were randomly selected until the target was achieved.

The inclusion criteria were as follows: mother–child pairs, with the child aged 5–6 years old, enrolled in a preschool under the Ministry of National Education in Samsun, accompanied by the mother, residing in Samsun, willing to participate, and providing informed consent.

Exclusion criteria were non-compliance with these criteria, refusal to participate, single-parent families, mothers who do not speak Turkish, families without internet access, children with a Body Mass Index Z-score (BAZ) < −1 (indicating risk of underweight), and children with chronic illnesses affecting growth.

### 2.4. Participant Flow and Data Cleaning Process

**Figure 1:**
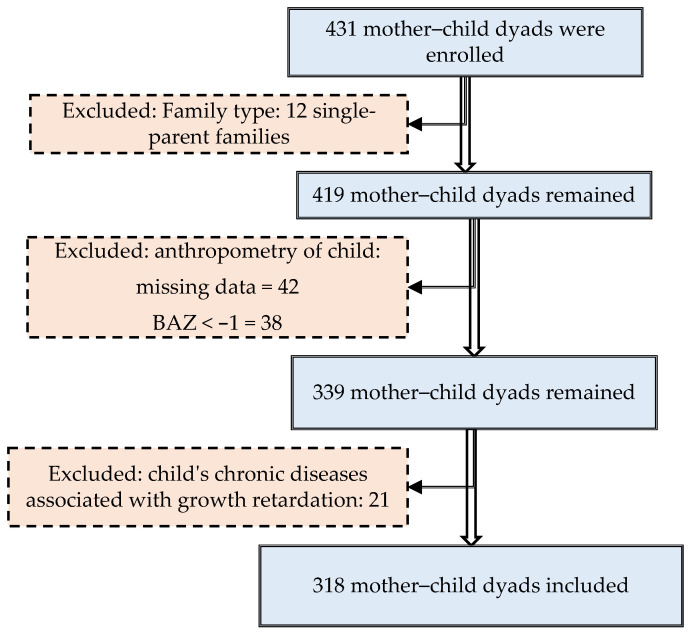
Participant flow diagram: illustrating selection and exclusion process.

A total of 512 mothers were invited to participate in this study, of which 431 agreed to participate. However, certain cases were excluded during the data cleaning process. Specifically, 12 mother–child dyads were excluded because the mothers were from single-parent or broken families, which could influence the mother’s perception of the child’s body size and eating behaviors. In these cases, the primary caregiver might not have been the participating mother, or the child might have been under the care of multiple caregivers. Additionally, 42 children had missing anthropometric data, 38 children had a BAZ below −1, and 21 children had chronic illnesses that could potentially affect growth, including asthma, epilepsy, PFAPA (Periodic Fever, Aphthous Stomatitis, Pharyngitis, and Adenitis), Type 1 Diabetes Mellitus (DM), and cerebral palsy. After these exclusions, the final analysis included 318 mother–child pairs (see Figure 1).

### 2.5. Measures

Data collection was initially conducted through Google Forms surveys for the mothers’ questionnaires. Following maternal consent, children’s height and weight were measured at their respective schools.

#### 2.5.1. Surveys

The surveys for this study were designed and distributed to participating mothers via Google Forms, enabling secure, efficient, and accessible online data collection. Links to the surveys were shared with mothers through their children’s teachers, ensuring accessibility and efficient data collection. This digital approach minimized transcription errors by automating data entry, thus streamlining data preparation for analysis. To maintain participant anonymity, no personally identifiable information (PII) was collected. Each mother and child were assigned a unique code, allowing for the accurate pairing of data from mother–child pairs without compromising confidentiality. These codes were essential for data alignment during analysis, preserving the integrity and validity of responses.

The survey consisted of four primary sections that assess socio-demographic characteristics, child eating behaviors, parenting attitudes, and maternal perceptions of child body size using a modified visual assessment tool.

The Socio-Demographic Characteristics section gathered key information, including parents’ and child’s age, parents’ education attainment and employment status, family structure (e.g., nuclear or extended), financial support status, resources consulted by the mother for educational and health-related decisions, primary caregivers supporting the mother, birth-related details (e.g., birth weight, prematurity, birth order), child’s history of hospital admissions, and duration of preschool education.

The Children’s Eating Behavior Questionnaire (CEBQ), developed by Wardle et al. [16], was used to evaluate various eating behaviors. This validated tool consists of 35 Likert-scale items (ranging from 1 = never to 5 = always) across eight subscales: food responsiveness, emotional overeating, emotional undereating, enjoyment of food, desire to drink, satiety responsiveness, slowness in eating, and food fussiness. These subscales assess a wide range of eating patterns and behavioral traits linked to risks such as obesity and eating disorders. The Turkish version of the CEBQ, adapted by Yılmaz et al. [39], demonstrated reliability, with Cronbach’s alpha values ranging from 0.61 to 0.86 across subscales. In our study, this tool was administered via Google Forms, enabling efficient and standardized data collection. Table 1 summarizes the percentile distribution and reliability (Cronbach’s alpha) of the CEBQ scores as calculated in our study. Among the CEBQ subscales, ’Enjoyment of Food’ displayed the highest reliability (α = 0.86), while ’Emotional Overeating’ and ’Emotional Undereating’ exhibited relatively lower reliability values (α = 0.67 and α = 0.69, respectively). These results confirm acceptable internal consistency across all subscales in the context of our sample.

Parenting attitudes were assessed using the Parental Attitude Scale (PAS), a validated tool designed for Turkish parents of young children [40]. The PAS comprises 46 Likert-scale items (ranging from 1 = never to 5 = always) and categorizes parenting styles into four groups: authoritative, authoritarian, overprotective, and permissive. As shown in Table 1, Authoritative Parenting emerged as the most reliable subscale (α = 0.91) and had the highest mean score (75.8), highlighting its predominance among the participating mothers. Conversely, ’Permissive Parenting’ showed the lowest reliability (α = 0.68), with a mean score of 20.0, indicating its less frequent preference among this population.

Maternal perceptions of child body size were evaluated using a modified visual assessment tool adapted from previous studies. Warschburger and Kröller [41] used silhouette scales to evaluate maternal perceptions of weight and associated health risks, reporting a 64.5% accuracy rate in identifying overweight silhouettes and highlighting the influence of maternal education on perception accuracy. Hager et al. [42] developed the Toddler Silhouette Scale, demonstrating robust validity and reliability measures, including content validity (κ = 0.710, *p* < 0.001) and concurrent validity (r = 0.633, *p* < 0.001). Similarly, Yalçın et al. [5] used a validated visual tool to assess maternal perception, reporting significant correlation between verbal and visual assessments (r = −0.620, *p* < 0.001). The tool consisted of seven gender-neutral images representing a range of weight percentiles, ensuring broad applicability across different contexts [5,41,42]. To confirm its clarity and suitability for the study population, the tool was pre-tested with a sample of 30 participants prior to the main study. The visual tool was integrated into the Google Forms survey, where mothers were asked to respond to the following question: “Which image best represents your child’s current size?” For analytical purposes, maternal responses were grouped into three categories: scores 1–3 were categorized as underweight, score 4 as normal weight, and scores 5–7 as overweight. This categorization enabled a structured analysis of maternal perceptions, offering valuable insights into how mothers perceive their child’s body size relative to their actual weight status.

#### 2.5.2. Anthropometric Measures

Children’s anthropometric measurements, including height and weight, were directly measured by the researcher to ensure data accuracy and consistency. Height was measured to the nearest 0.1 cm using a wall-mounted paper stadiometer, and weight was recorded to the nearest 0.1 kg using Xiaomi Mi Body Composition Scale 2 (Xiaomi Inc., Beijing, China). All measurements were conducted under standardized conditions, with children in light clothing and without shoes. The Weight-for-Age Z-score (WAZ), Height-for-Age Z-score (HAZ), and BMI-for-age Z-score (BAZ) according to age and sex were subsequently calculated using the World Health Organization’s AnthroPlus software version 1.0.4. BAZ values were categorized in accordance with the World Health Organization’s standards for children aged 5–19 years old [43]. Scores below −2 SD were classified as thinness, while those between −2 SD and −1 SD indicated a risk of underweight. Scores ranging from −1 SD to +1 SD were considered normal weight. Values between >+1 SD and ≤+2 SD represented overweight status, and scores above +2 SD were categorized as obesity.

### 2.6. Variables

The primary independent variables included in this study were family characteristics (parents’ age, education level, and employment status; family structure [nuclear or extended]; and financial support status), parenting attitudes (authoritative, authoritarian, overprotective, and permissive styles), sources of information and caregiving support, birth and health history of the child, years spent in preschool education, and the subscales of the CEBQ.

The dependent variables were maternal estimation accuracy (derived from BMI Z-scores) and the maternal perception of the child’s body size. The maternal perceptions of child weight were assessed using a visual tool, and the actual weight category was matched with the maternally perceived child size. These were coded into three categories:

- Accurate Estimation: Maternal perception aligned with the child’s actual weight category.
- Underestimation: Maternal perception was smaller than the child’s actual weight category.
- Overestimation: Maternal perception was larger than the child’s actual weight category.

For the final analysis, three cases of underestimation were excluded to ensure statistical robustness. The remaining data were categorized into two groups for comparison: accurate estimation and overestimation.

### 2.7. Bias

Several measures were implemented to minimize potential sources of bias and ensure the validity of the study findings. Data collection was conducted via Google Forms, which reduced social desirability bias by allowing participants to respond more candidly online. Anonymity was maintained by assigning unique codes to each mother and child pair, promoting honest responses. All anthropometric measurements were conducted by a single trained researcher to ensure consistency and minimize inter-observer variability. Additionally, standardized protocols were followed for measuring children’s height and weight, which helped reduce potential measurement bias. Although stratified sampling was employed to ensure representativeness across socio-economic strata, the exclusion of certain groups, such as single-parent families or children with chronic illnesses, may have introduced selection bias. These exclusions were made because chronic illnesses can influence growth patterns, potentially altering maternal perceptions of weight. Similarly, single-parent families or those with shared custody arrangements might experience differences in maternal perceptions due to unique family dynamics and caregiving structures. By excluding these groups, this study aimed to focus on maternal perceptions under more typical family conditions and reduce confounding effects.

Validated tools, such as the CEBQ and PAS, were confirmed with high Cronbach’s alpha values, supporting the robustness of the assessments. Finally, a pre-test of the visual tool helped ensure clarity and prevent interpretation bias among participants. By implementing these measures, this study aimed to mitigate potential biases and strengthen the reliability of its conclusions.

### 2.8. Statistical Analysis

Data were analyzed using IBM SPSS Statistics for Windows, Version 23.0 (IBM Corp., Armonk, NY, USA). Descriptive statistics were presented as frequencies and percentages for categorical variables and as the mean ± standard deviation or median with interquartile ranges (IQRs) for continuous variables, depending on the data distribution. The distribution characteristics of continuous variables were assessed using histograms, kurtosis, skewness, and the Kolmogorov–Smirnov test.

The primary variable of interest was the accuracy of the maternal assessment of the child’s body size (accurate vs. underestimate). Differences in child characteristics and maternal–child variables between the “accurate” and “underestimate” groups were evaluated using the independent samples *t*-test or Mann–Whitney U test for continuous variables, based on distribution. Differences in categorical variables were assessed using the chi-square test or Fisher’s exact test, as appropriate.

Variables with a *p*-value < 0.2 in univariate analysis were included in a multiple logistic regression model to examine their associations with accurate maternal assessments of normal weight, overweight, and combined (overall) child groups. Additionally, interquartile ranges (IQRs) were calculated for each subscale of the Children’s Eating Behavior Questionnaire (CEBQ), and the association between each unit change in subscale scores and maternal assessment accuracy was analyzed. Adjusted odds ratios (AORs) with 95% confidence intervals (CIs) were reported.

A *p*-value < 0.05 was considered statistically significant.

## 3. Results

### 3.1. General Characteristics of Participants

This study involved 318 mother–child pairs. Mothers had a mean age of 34.7 ± 5.0 years, while fathers were slightly older at 37.9 ± 5.8 years. Most mothers (81.1%) and fathers (81.8%) had completed at least 12 years of formal education. Approximately one-third of the mothers (30.8%) were employed. Regarding health, 8.2% of mothers reported having a chronic illness, while 11.9% were taking medication during the study period. Most families were nuclear (93.1%), and a minority (6.6%) relied on financial support either from the government or private institutions.

Children had a mean age of 5.5 ± 0.3 years old, with a slight majority being male (51.3%). Birth order data indicated that 51.9% were firstborns, and 20.8% were only children. The majority (83.6%) were born at a normal weight (2.5–3.9 kg), with 8.5% born at a low birth weight (<2.5 kg). Prematurity was reported in 12.3% of the children. A substantial proportion (34.9%) had a history of hospital admissions, and approximately one-third (33.0%) were in their first year of preschool (Table 2).

Primary caregivers assisting mothers included fathers (79.9%), grandparents (33.0%), and elder siblings (10.1%). Few families relied on nannies or other caregivers (1.6% each). The most frequently consulted sources for educational information were teachers (76.7%) and books (47.8%), while healthcare advice was primarily obtained from pediatricians (86.5%) and family physicians (70.8%).

The scores of maternal parenting styles are given in Table 2.

Children’s eating behaviors included a median food responsiveness score of 9 [6–11], a mean enjoyment of food score of 15.4 ± 4.8, and an average satiety responsiveness score of 22.6 ± 5.5, highlighting differences in response to food cues. Additional behaviors such as emotional overeating (median: 6 [4–8]), desire to drink (mean: 8.1 ± 3.5), slowness in eating (mean: 10.6 ± 4.0), emotional undereating (mean: 11.5 ± 3.8), and food fussiness (median: 6.5 [5–9]) (Table 2).

Among the mothers surveyed, 59.7% underestimated their child’s weight, with only 39.3% accurately assessing their child’s weight status. Overestimation was rare, occurring in just 0.9% of cases (Table 3). Due to the small size of the overestimation subgroup, meaningful statistical analysis could not be performed, and these cases were not included in the subgroup comparisons.

**Figure 2:**
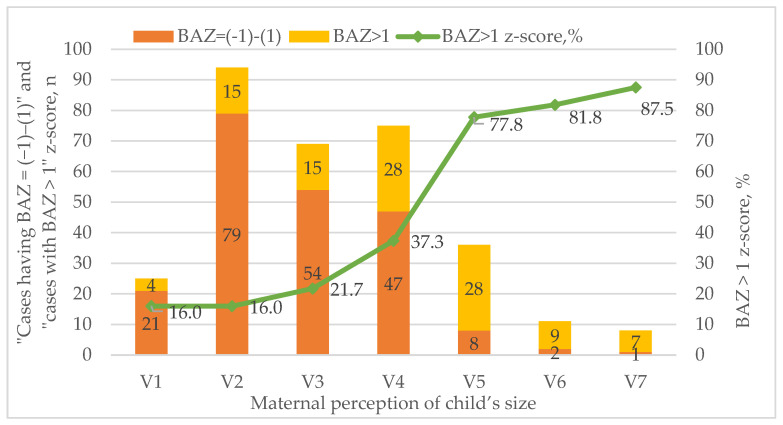
Association between maternal perception of child size and BAZs.

Maternal perceptions of children’s weight status, assessed through a visual tool, were compared with these objective measures to evaluate accuracy and trends in underestimation or overestimation (Figure 2). Maternal perceptions of child size were categorized into three groups, underweight (V1–V3), normal weight (V4), and overweight (V5–V7), based on the distribution pattern and the slope of the green line representing obesity rates (BAZ > +1). For the underweight category (V1–V3), obesity rates ranged from 16% to 21.7%. In the normal weight category (V4), the obesity rate was 37.3%. For the overweight category (V5–V7), obesity rates ranged from 77.8% to 87.5%.

### 3.2. Comparative Analysis Between Maternal Accurate and Underestimation Groups

An analysis of familial characteristics revealed that maternal or paternal age, education level, maternal employment status, and maternal health conditions showed no statistically significant association with accurate maternal estimation. However, family structure was associated with accurate estimation, with mothers in nuclear families more likely to underestimate their child’s weight (62.1%) compared to those in extended families (36.4%), with a statistically significant difference (*p* = 0.017, Table 4).

No significant differences in parenting style scores were observed between accurate estimators and underestimators across all styles, including authoritative, authoritarian, overprotective, and permissive parenting (*p* > 0.05, Table 4).

While no significant differences were observed in the educational information sources consulted by mothers, health information sources revealed a notable difference in accurate estimation (*p* > 0.05). Mothers who consulted relatives for health-related advice were significantly more likely to underestimate their child’s weight (74.6%) compared to those who did not (56.5%, *p* = 0.007, Table 4). This suggests that reliance on non-professional health advice may contribute to the maternal underestimation of weight status.

The analysis of child characteristics revealed a significant association between birth order and maternal estimation accuracy. The mothers of firstborn children were more likely to estimate accurately (44.2%) compared to those with later-born children, where underestimation was higher (65.3%), with a significant difference (*p* = 0.024). However, no significant associations were found for child’s age, sex, gestational duration, birth weight, or hospital admission history and years of preschool education (*p* > 0.05, Table 5).

Maternal estimation accuracy also showed a strong relation with BMI-for-age Z-scores (BAZs). Among children with a BAZ ≤ 1 (normal weight), 52.2% were accurately estimated, whereas underestimation was prevalent (84.9%) among children with a BAZ > 1 (overweight) (*p* < 0.001). This highlights a critical trend where maternal perception becomes less accurate as the child’s weight increases, potentially delaying the recognition of weight-related issues (Table 5).

Mothers with accurate perceptions reported higher scores in food responsiveness (median: 9 [7–12] vs. 5 [4–8], *p* = 0.028) and the enjoyment of food (mean: 16.3 ± 4.8 vs. 14.6 ± 4.7, *p* = 0.003). Conversely, underestimators reported higher satiety responsiveness (mean: 23.2 ± 5.7 vs. 21.9 ± 5.2, *p* = 0.042) and slowness in eating (mean: 11.2 ± 4.2 vs. 9.9 ± 3.6, *p* = 0.004). These findings suggest that children’s responsiveness to food cues and eating patterns may influence maternal perception, with underestimation potentially linked to less observable cues such as satiety and eating speed. No significant associations were identified for other child characteristics, including age, sex, gestational duration, birth weight, breastfeeding duration, or hospital admission history. Similarly, years of preschool education showed no significant association (*p* > 0.05, Table 5).

### 3.3. Multiple Logistic Regression Analysis of Factors Influencing Maternal Perception in Normal Weight, Overweight, and Overall Child Groups

A logistic regression analysis examined factors influencing maternal perception in normal weight, overweight, and combined (overall) child groups (Table 6).

In the normal weight group, emotional overeating was associated with a 2.38-fold increase in accurate perception likelihood (OR = 0.42, 95% CI = 0.20–0.88, *p* = 0.021). Additionally, being an only child increased the likelihood of accurate maternal perception compared to children with siblings (OR = 0.37, 95% CI = 0.14–0.99, *p* = 0.047). Consulting relatives for educational advice also improved perception accuracy (OR = 11.16, 95% CI = 1.05–118.77, *p* = 0.046).

In the overweight group, employed mothers were less likely to underestimate their child’s weight compared to non-employed mothers (OR = 0.05, 95% CI = 0.00–0.56, *p* = 0.015). Higher scores in the enjoyment of food (OR = 0.03, 95% CI = 0.00–0.35, *p* = 0.005) and satiety responsiveness (OR = 0.09, 95% CI = 0.01–0.67, *p* = 0.019) reduced underestimation likelihood. First-year school attendance was similarly linked to improved perception accuracy (OR = 0.50, 95% CI = 0.25–0.98, *p* = 0.045).

In the overall group, slow eating was associated with a higher likelihood of underestimation (OR = 1.62, 95% CI = 1.04–2.54, *p* = 0.033). Conversely, first-year school attendance (OR = 0.54, 95% CI = 0.30–0.97, *p* = 0.041) and higher BAZs (OR = 12.16, 95% CI = 5.80–25.51, *p* < 0.001) were associated with improved accuracy in weight perception.

## 4. Discussion

### 4.1. Maternal Perceptions, Ecological Influences, and Implications for Childhood Obesity Prevention

This study provides a comprehensive analysis of the maternal perceptions of child weight status, identifying critical trends and associations with socio-cultural, familial, and child-specific factors. Grounded in Bronfenbrenner’s Ecological Systems Theory, the findings highlight the multi-layered determinants of maternal underestimation, a significant global barrier to addressing childhood obesity [26,27,28].

### 4.2. The Underestimation of Child Weight: A Global Challenge

Our findings revealed that 59.7% of mothers underestimated their child’s weight, with underestimation reaching 84.9% among overweight children. This trend mirrors findings from diverse cultural settings, emphasizing its pervasive nature. This finding is consistent with studies from diverse cultural settings. For instance, traditional preferences for heavier child body types in Latin America and lower socio-economic groups contribute to frequent misperceptions [8,44]. Similarly, cultural norms associating larger body sizes with health and well-being are evident in Türkiye, where socio-economic disparities and marginalized populations further complicate maternal awareness [6]. The persistence of underestimation in high-resource settings, such as the Netherlands, suggests that addressing maternal misperception requires more than public awareness campaigns; it necessitates tackling implicit biases and societal norms [45,46]. Aligning with frameworks like the SDGs and the UNICEF Nurturing Care Framework, equitable interventions can bridge these gaps and empower families [2,24,25,47].

### 4.3. Microsystem: The Family Context

At the microsystem level, nuclear family structures were associated with higher underestimation rates (62.1%) compared to extended families (36.4%, *p* = 0.017). This discrepancy suggests that the absence of diverse familial perspectives in nuclear families may limit opportunities for maternal discussions or comparisons regarding a child’s weight status. Extended family members, particularly grandparents, might provide additional input that helps mothers evaluate their child’s body size more accurately. Birth order also played a significant role: firstborn children were more accurately perceived than their later-born counterparts, supporting Social Comparison Theory. First-time mothers, likely relying more on professional guidance and direct observations, exhibited enhanced perception accuracy. This finding aligns with prior research suggesting that maternal perceptions are shaped by early parenting experiences and external influences [31,32].

Child-specific factors also influenced perception. Emotional overeating in the normal weight group increased the likelihood of accurate perception by 2.38-fold (OR = 0.42, 95% CI = 0.20–0.88, *p* = 0.021). Conversely, low satiety responsiveness and slow eating were associated with underestimation, reflecting maternal misinterpretations of appetite cues. These findings align with the Bidirectional Parent–Child Interaction Model, which posits that children’s eating behaviors directly influence parental perceptions and feeding practices [29]. Contrary to expectations, no significant associations were found between parenting styles and maternal perception accuracy. This may suggest that socio-cultural norms, educational attainment, and exposure to health messaging exert a stronger influence than parenting style alone. These findings emphasize the complexity of maternal perception, highlighting the need for future longitudinal studies to explore how these factors interact over time to shape maternal awareness.

### 4.4. Mesosystem: Interactions with Schools and Healthcare Providers

The mesosystem level highlights the role of schools and healthcare providers in shaping maternal perceptions. Paradoxically, first-year school attendance was associated with higher underestimation (OR = 0.54, 95% CI = 0.30–0.97, *p* = 0.041), potentially due to the maternal normalization of higher weights when comparing their child with peers. This phenomenon, explained by Social Comparison Theory, highlights the potential for school environments to unintentionally perpetuate misperceptions. Consulting relatives for educational advice was positively associated with accurate perception in the normal weight group (OR = 11.16, 95% CI = 1.05–118.77, *p* = 0.046). This underscores the nuanced influence of non-professional sources. However, reliance on relatives for healthcare advice warrants further exploration, as it may perpetuate cultural biases and misperceptions.

### 4.5. Macrosystem: Societal Norms and Global Frameworks

At the macrosystem level, societal norms associating higher child body weights with resilience or prosperity remain entrenched, complicating public health efforts. Systemic interventions are required to challenge these narratives and promote accurate maternal perceptions. Global frameworks such as the UNICEF Nurturing Care Framework and the WHO’s Obesity Prevention Strategies advocate for multifaceted approaches to tackle childhood obesity. These frameworks align with the SDGs, particularly Goal 3, which emphasizes health equity and well-being. Targeting socio-economically disadvantaged families through culturally sensitive education and leveraging community resources can bridge the gap between maternal perceptions and clinical realities, ensuring that no child is left behind [2,6,36,48].

### 4.6. Responsive Feeding and Nurturing Care: A Path Forward

This study underscores the relevance of responsive feeding as a key component of the Nurturing Care Framework. Mothers who accurately perceived their child’s weight reported higher levels of food responsiveness and enjoyment of food in their children, consistent with research linking child-centered feeding practices to better maternal awareness [25]. Conversely, mothers who underestimated their child’s weight reported lower satiety responsiveness and higher emotional overeating in their children. These findings emphasize the importance of moving from restrictive parent-centered feeding to responsive child-centered feeding approaches, that respect children’s hunger and satiety cues. Low satiety responsiveness was associated with the maternal underestimation of child weight, potentially reflecting the maternal misinterpretation of appetite cues. According to the Bidirectional Parent–Child Interaction Model, children’s eating behaviors can shape maternal perceptions and feeding practices, reinforcing these misperceptions. While our study did not directly assess responsive feeding practices, the CEBQ results highlight their potential in improving parental awareness of children’s nutritional status and reducing underestimation [2,16,39]. By respecting children’s hunger and satiety cues, responsive feeding practices can improve parental awareness of children’s nutritional status and reduce underestimation. This aligns with recommendations by UNICEF and WHO for promoting healthy growth and development in early childhood [2,16,25,49].

### 4.7. Strengths and Limitations

This study provides valuable insights into the maternal perceptions of child weight status and their associations with familial and child-specific factors within the socio-cultural context of Samsun, Türkiye. By including participants from all socio-economic development levels (SEGE) in Samsun, this study ensures representation across diverse socio-economic contexts, enhancing its generalizability within the region. Moreover, its focus on preschool-aged children provides critical data on maternal awareness during a pivotal stage for shaping long-term health behaviors. The inclusion of maternal reports on children’s eating behaviors offers a novel, child-centered perspective, shifting the focus from parental practices to children’s dietary patterns. This approach provides fresh insights into the interplay between maternal perceptions and body size recognition, an area previously underexplored in research.

Despite these strengths, several limitations should be acknowledged. The cross-sectional design precludes causal inferences, and future longitudinal studies are necessary to explore how maternal misperceptions influence child health outcomes over time. Additionally, reliance on maternal self-reported data introduces potential biases, such as social desirability bias, which may affect responses about sensitive topics like parenting styles. Although confidentiality measures were implemented, these biases cannot be fully excluded.

The assessment of parenting styles was based on maternal self-reports at a single time point, limiting the ability to capture the dynamic and evolving nature of parenting behaviors. Longitudinal research could provide a more comprehensive understanding of how these styles influence maternal perception accuracy and child outcomes over time. Furthermore, maternal BMI, a known factor influencing weight perceptions, was not collected, representing a gap in the dataset. Including maternal BMI in future research could provide insights into how mothers’ own weight status affects their perceptions of their children’s weight.

This study excluded fathers. However, paternal parenting styles and involvement likely impact child weight perceptions. The decision to focus solely on mothers aimed to create a homogeneous sample; however, future studies including fathers would provide a more holistic perspective. Similarly, the exclusion of children from private preschools or those not enrolled in any early education program introduces potential selection bias, limiting the generalizability of the findings to children in alternative educational settings. Certain groups, such as single-parent families and children with chronic illnesses, were excluded to reduce confounding factors. While this decision was made with the aim to focus on maternal perceptions under typical family conditions, these exclusions may overlook unique dynamics that influence perceptions. Chronic illnesses, for instance, affect growth patterns and maternal views on weight, while single-parent families bring distinctive caregiving challenges.

The small sample size of mothers who overestimated their children’s weight restricted subgroup analyses, underscoring the need for larger and more diverse sample sizes in future research. Additionally, this study’s findings are specific to the socio-cultural and economic context of Samsun, and maternal perceptions may differ across regions with varying cultural norms, socio-economic profiles, and healthcare access. Expanding research to broader national or global scales is necessary to capture these variations and inform culturally tailored public health interventions.

Anthropometric measurements, such as skinfold thickness, were not included, limiting the ability to assess detailed body fat distribution. Including such measurements in future studies could enhance the accuracy of obesity classification and deepen the understanding of maternal perception in relation to objective weight measures.

By addressing these limitations through methodological enhancements and expanded participant diversity, future research can build on the current study’s findings and contribute to a more nuanced understanding of maternal perceptions and their role in preventing childhood obesity. Despite these limitations, this study makes a significant contribution by highlighting the multifaceted factors influencing the maternal perceptions of child weight status. Its novel focus on children’s eating behaviors, as perceived by mothers, provides a foundation for future research and the development of targeted strategies to prevent childhood obesity.

## 5. Conclusions

This study demonstrates the pervasive nature of the maternal underestimation of child weight status, particularly among overweight children, with rates as high as 84.9%. These findings highlight critical barriers to early intervention for childhood obesity. While no significant associations were observed between parenting styles and maternal perception accuracy, child-specific factors, such as food responsiveness and the enjoyment of food, emerged as influential determinants. This emphasizes the need for targeted early education programs for mothers that promote the awareness of healthy weight standards and encourage the adoption of responsive feeding practices as part of nurturing care.

To advance this field, future research should focus on longitudinal studies examining the long-term impact of maternal perceptions and feeding practices on child weight outcomes. Additionally, exploring how diverse cultural and socio-economic contexts shape these dynamics will be crucial. Public health strategies should align with global frameworks, such as the Nurturing Care Framework and the Sustainable Development Goals, to ensure culturally sensitive and inclusive interventions. These efforts can empower parents to address childhood obesity effectively, fostering healthier growth and development outcomes for children worldwide.

## Figures and Tables

**Table 1:** The percentile distribution of the Children’s Eating Behavior Questionnaire scores for preschoolers and the Parental Attitude Scale scores for their mothers.

	Mean	Percentile Values							Number of Items	Cronbach’s Alpha
		5	15	25	50	75	85	95		
**Children’s Eating Behavior Questionnaire**										
Food responsiveness	9.3	5	5	6	9	11	13	17	5	0.75
Emotional overeating	6.3	4	4	4	6	8	9	11	4	0.67
Enjoyment of food	15.4	7	10	12	16	19	20	24	5	0.86
Desire to drink	8.1	3	4	5	8	11	12	15	3	0.79
Satiety responsiveness	22.6	13	17	19	22	27	29	32	7	0.74
Slowness in eating	10.6	5	6	8	10	13	15	19	4	0.77
Emotional undereating	11.5	5	8	9	11	14	16	18	4	0.69
Food fussiness	7.1	3	3	5	7	9	11	13	3	0.78
**Parental Attitude Scale**										
Authoritative parenting	75.8	57	69	73	78	82	84	85	17	0.91
Authoritarian parenting	19.2	11	13	15	18	22	25	30	11	0.78
Overprotective parenting	33.3	21	26	29	34	38	41	44	9	0.79
Permissive parenting	20.0	12	14	16	20	23	25	29	9	0.68

**Table 2:** General characteristics, n = 318.

Familial Characteristics	N (%) Mean ± SD	Child Characteristics	N (%) Mean ± SD
Mother’s age	34.7± 5.0	Birth order, first child	165 (51.9)
Father’s age	37.9± 5.8	Only child, yes	66 (20.8)
Maternal education, ≥12 years	258 (81.1)	Child age	5.5 ± 0.3
Paternal education, ≥12 years	260 (81.8)	Sex, male	163 (51.3)
Working mother, yes	98 (30.8)	Gestation duration < 37 weeks	39 (12.3)
Mother’s chronic disease status, yes	26 (8.2)	Birth weight, kg	
Mother’s medication use, yes	38 (11.9)	<2.5	27 (8.5)
Family type, nuclear	296 (93.1)	2.5–3.9	266 (83.6)
Financial support, yes	21 (6.6)	≥4.0	25 (7.9)
Residence (SEGE Index)		Breastfeeding duration	
Level 1	93 (29.2)	<6 months	66 (20.8)
Level 2	76 (23.9)	6–11 months	52 (16.4)
Level 3	65 (20.4)	12–23 months	98 (30.8)
Level 4	46 (14.5)	≥24 months	102 (32.1)
Level 5	38 (11.9)	Child’s hospital admissions, yes	111 (34.9)
		Years of preschool education	
**Sources Consulted by the Mother**		First year	105 (33.0)
***Educational Information Sources***		Second year	164 (51.6)
Teachers	244 (76.7)	≥Third year	49 (15.4)
Books	152 (47.8)	**Children’s Eating Behavior**	
Online sources	147 (46.2)	Food responsiveness	9 (6–11)
Relatives	92 (28.9)	Emotional overeating	6 (4–8)
Friends	174 (54.7)	Enjoyment of food	15.4 ± 4.8
***Health Information Sources***		Desire to drink	8.1 ± 3.5
Pediatricians	275 (86.5)	Satiety responsiveness	22.6 ± 5.5
Family physicians	225 (70.8)	Slowness in eating	10.6 ± 4.0
Family health center nurses	53 (16.7)	Emotional undereating	11.5 ± 3.8
Healthcare professionals	305 (95.9)	Food fussiness	6.5 (5–9)
Books	21 (6.6)	**Maternal Parenting Style**	
Online resources	71 (22.3)	Authoritative parenting	78 (73–82)
Relatives	68 (21.4)	Authoritarian parenting	18 (15–22)
Friends	50 (15.7)	Overprotective parenting	34 (29–38)
		Permissive parenting	20 ± 5.2

**Table 3:** Anthropometric measurements of children and perceptions of their mothers, n = 318.

Anthropometric Measurements	N (%)	Perception/Accurate Estimation	N (%)
**Weight-for-Age Z-score**		**Maternal perception of child’s size**	
Median [25–75 percentile]	0.46 [(−0.23)–(1.27)]	Underweighted, 1–3	188 (59.1)
>(2) z-score	32 (10.1)	Normal weighted, 4	75 (23.6)
**Height-for-Age Z-score**		Overweighted, 5–7	55 (17.3)
Median [25–75 percentile]	0.18 [(0.52)–(0.89)]		
<(−1)–≥(−2) z-score (at risk of stunted)	35 (11.0)		
<(−2) z-score (stunted)	2 (0.6)	**Accurate estimation compared to BAZ**	
**BMI-for-Age Z-score**		Overestimation	3 (0.9)
Median [25–75 percentile]	0.47 [(0.17)–(1.31)]	Accurate estimation	125 (39.3)
>1–≤2 z-score (overweight)	68 (21.4)	Underestimation	190 (59.7)
>2 z-score (obese)	38 (12.0)		

**Table 4:** Differences in familial characteristics, information sources, and maternal parenting styles between accurate and underestimation groups (n = 315).

		Accurate Estimation *	Underestimation *	*p*
**n**		125	190	
**Familial characteristics**				
Mother’s age		34.5 ± 4.5	34.9 ± 5.3	0.485
Father’s age		34.5 ± 5.7	34.9 ± 5.3	0.312
Maternal education	≥12 years	104 (40.6)	152 (59.4)	0.476
	<12 years	21 (35.6)	38 (64.4)	
Paternal education	≥12 years	102 (39.5)	156 (60.5)	0.909
	<12 years	23 (40.4)	34 (59.6)	
Family type	Nuclear	111 (37.9)	182 (62.1)	0.017
	Extended	14 (63.6)	8 (36.4)	
Mother’s chronic disease status	Yes	9 (34.6)	17 (65.4)	0.581
	No	116 (40.1)	173 (59.9)	
Mother employed	Yes	46 (47.4)	51 (52.6)	0.061
	No	79 (36.2)	139 (63.8)	
Financial/social support	Yes	5 (23.8)	16 (76.2)	0.124
	No	120 (40.8)	174 (59.2)	
**Educational Information Sources of the Mother**				
Teachers	Yes	101 (41.7)	141 (58.3)	0.175
	No	24 (32.9)	49 (67.1)	
Books	Yes	62 (41.6)	87 (58.4)	0.508
	No	63 (38.0)	103 (62.0)	
Online sources	Yes	59 (40.1)	88 (59.9)	0.878
	No	66 (39.3)	102 (60.7)	
Relatives	Yes	30 (32.6)	62 (67.4)	0.099
	No	95 (42.6)	128 (57.4)	
Friends	Yes	68 (39.3)	105 (60.7)	0.880
	No	57 (40.1)	85 (59.9)	
**Health Information Sources of the Mother**				
Healthcare professionals	Yes	122 (40.4)	180 (59.6)	0.211
	No	3 (23.1)	10 (76.9)	
Books	Yes	6 (28.6)	15 (71.4)	0.281
	No	119 (40.5)	175 (59.5)	
Online resources	Yes	25 (35.2)	46 (64.8)	0.382
	No	100 (41.0)	144 (59.0)	
Relatives	Yes	17 (25.4)	50 (74.6)	0.007
	No	108 (43.5)	140 (56.5)	
Friends	Yes	16 (32.0)	34 (68.0)	0.226
	No	109 (41.1)	156 (58.9)	
**Maternal parenting style**				
Authoritative parenting		79 [73.5–83]	78 [73–82]	0.587
Authoritarian parenting		18 [15–22]	19 [15–22.3]	0246
Overprotective parenting		34 [30–38]	33 [28–38]	0.531
Permissive parenting		20.5 ± 5.6	19.8 ± 4.8	0.226

* Mean ± SD or median [25th–75th percentile] or N (row percentage).

**Table 5:** Differences in child characteristics and children’s eating behavior styles between accurate and underestimation groups (n = 315).

		Accurate Estimation *	Underestimation *	*p*
Birth order	First child	73 (44.2)	92 (55.8)	0.024
	≥2	52 (34.7)	98 (65.3)	
Only child	Yes	31 (47.0)	35 (53.0)	0.083
	No	94 (37.8)	155 (62.2)	
Child’s age		5.4 ± 0.3	5.5 ± 0.3	0.130
Sex	Female	65 (42.5)	88 (57.5)	0.323
	Male	60 (37.0)	102 (63.0)	
Gestational duration	<37 weeks	12 (30.8)	27 (69.2)	0.224
	≥37 weeks	113 (40.9)	163 (59.1)	
Birth weight, kg	≥2500	114 (39.6)	174 (60.4)	0.906
	<2500	11 (40.7)	16 (59.3)	
Breastfeeding duration	<6 months	26 (39.4)	40 (60.6)	0.412
	6–11 months	15 (29.4)	36 (70.6)	
	12–23 months	41 (42.3)	56 (57.7)	
	≥24 months	43 (42.6)	58 (57.4)	
Child’s hospital admissions	Yes	44 (40.7)	64 (59.3)	0.782
	No	81 (39.1)	126 (60.9)	
Years of preschool education	First year	50 (47.6)	55 (52.4)	0.126
	≥Second year	75 (35.7)	135 (64.3)	
**BMI-for-Age Z-score**	≤1	109 (52.2)	100 (47.8)	<0.001
	>1	16 (15.1)	90 (84.9)	
**Children’s Eating Behavior**				
Food responsiveness		9 [7–12]	5 [4–8]	0.028
Emotional overeating		6 [4–8]	5 [4–8]	0.056
Enjoyment of food		16.3 ± 4.8	14.6 ± 4.7	0.003
Desire to drink		7.8 ± 3.3	8.3 ± 3.6	0.247
Satiety responsiveness		21.9 ± 5.2	23.2 ± 5.7	0.042
Slowness in eating		9.9 ± 3,6	11.2 ± 4.2	0.004
Emotional undereating		11.4 ± 3.8	11.7 ± 3.8	0.513
Food fussiness		7 [5–9]	6 [5–9]	0.586

* Mean ± SD or median [25th–75th percentile] or N (row percentage).

**Table 6:** Multiple logistic regression analysis of factors influencing maternal underestimation in normal weight, overweight, and overall child groups.

	Normal			Overweight			Overall		
	AOR	95% CI	*p*	AOR	95% CI	*p*	AOR	95% CI	*p*
Birth Order (Firstborn vs. Others)	0.55	0.27–1.12	0.098	0.09	0.01–1.22	0.070	0.55	0.30–1.03	0.062
Only Child (Only vs. Siblings)	0.37	0.14–0.99	0.047	7.03	0.64–76.88	0.110	0.63	0.29–1.36	0.237
First Year in School vs. Second Year or More	0.50	0.25–0.98	0.045	0.12	0.02–0.93	0.042	0.54	0.30–0.97	0.041
Family Structure (Extended vs. Nuclear)	0.72	0.22–2.37	0.589	0.46	0.02–8.77	0.602	0.48	0.17–1.35	0.163
Working Mother (Yes vs. No)	0.95	0.49–1.85	0.873	0.05	0.00–0.56	0.015	0.66	0.37–1.20	0.175
Financial/Social Support (Yes vs. No)	0.68	0.16–2.96	0.605	0.49	0.01–18.75	0.701	0.78	0.24–2.57	0.684
Consulting Relatives for Education (Yes vs. No)	11.16	1.05–118.77	0.046	0.61	0.02–22.71	0.787	4.68	0.88–24.85	0.070
Consulting Teachers for Education (Yes vs. No)	0.57	0.28–1.19	0.136	2.79	0.42–18.53	0.287	0.88	0.46–1.68	0.699
BMI-for-Age Z-score (BAZ 1 vs. 0)							12.16	5.80–25.51	0.000
Food Responsiveness, for each IQR increase	1.29	0.65–2.58	0.469	0.62	0.18–2.12	0.444	0.72	0.43–1.20	0.204
Emotional Overeating, for each IQR increase	0.42	0.20–0.88	0.021	2.13	0.57–8.00	0.263	0.81	0.47–1.40	0.452
Enjoyment of Food, for each IQR increase	1.01	0.50–2.03	0.984	0.03	0.00–0.35	0.005	0.67	0.37–1.23	0.200
Satiety Responsiveness, for each IQR increase	1.32	0.74–2.37	0.347	0.09	0.01–0.67	0.019	0.95	0.57–1.58	0.839
Slow Eating, for each IQR increase	1.54	0.92–2.60	0.102	1.79	0.45–7.05	0.406	1.62	1.04–2.54	0.033

IQR: interquartile range.

## Data Availability

The data presented in this study are available upon request from the corresponding author due to ethical considerations.
